# Wi-AM: Enabling Cross-Domain Gesture Recognition with Commodity Wi-Fi

**DOI:** 10.3390/s24051354

**Published:** 2024-02-20

**Authors:** Jiahao Xie, Zhenfen Li, Chao Feng, Jingzhi Lin, Xianjia Meng

**Affiliations:** School of Information Science and Technology, Northwest University, Xi’an 710127, China; xiejiahao@stumail.nwu.edu.cn (J.X.); lizhenfen@stumail.nwu.edu.cn (Z.L.); chaofeng@stumail.nwu.edu.cn (C.F.); jzLinn@126.com (J.L.)

**Keywords:** domain adaptation, deep learning, gesture recognition, Wi-Fi, wireless sensor

## Abstract

RF-based gesture recognition systems outperform computer vision-based systems in terms of user privacy. The integration of Wi-Fi sensing and deep learning has opened new application areas for intelligent multimedia technology. Although promising, existing systems have multiple limitations: (1) they only work well in a fixed domain; (2) when working in a new domain, they require the recollection of a large amount of data. These limitations either lead to a subpar cross-domain performance or require a huge amount of human effort, impeding their widespread adoption in practical scenarios. We propose Wi-AM, a privacy-preserving gesture recognition framework, to address the above limitations. Wi-AM can accurately recognize gestures in a new domain with only one sample. To remove irrelevant disturbances induced by interfering domain factors, we design a multi-domain adversarial scheme to reduce the differences in data distribution between different domains and extract the maximum amount of transferable features related to gestures. Moreover, to quickly adapt to an unseen domain with only a few samples, Wi-AM adopts a meta-learning framework to fine-tune the trained model into a new domain with a one-sample-per-gesture manner while achieving an accurate cross-domain performance. Extensive experiments in a real-world dataset demonstrate that Wi-AM can recognize gestures in an unseen domain with average accuracy of 82.13% and 86.76% for 1 and 3 data samples.

## 1. Introduction

Gesture recognition technology provides an intelligent and friendly way for multimedia devices to interact, and it is being applied in fields such as healthcare [1], traffic [2], smart homes [3], and more. This technology allows for smarter and friendlier interactions between people and appliances. To illustrate this, in IoT-enabled households, one could wave a hand to change the TV program in a contact-free way. In supermarkets, customers can purchase goods without using self-service devices. Traditional solutions for gesture recognition either require wearable devices [4,5,6] or the installation of cameras [7,8,9]. Although promising, these methods incur uncomfortable user experiences or invade user privacy. In recent years, wireless sensing technologies [10,11,12,13,14,15] are emerging as a promising alternative to achieve device-free privacy-preserving gesture recognition due to their device-free and non-intrusive nature. The key to wireless sensing technology is that the signal transmission process, through reflection or refraction, will record the human body movement information; different movement change patterns will cause different signals to change, so we can infer the human body movement information from the signal changes. Due to the non-directly observable nature of wireless signals, unlike directly visible videos or images, they can protect user privacy. Extensive recent efforts with various wireless signals, such as Wi-Fi [16,17,18], RFID [19,20,21], acoustic [22,23,24] and mmWave [25,26,27], have been devoted to inferring gestures. Wi-Fi devices can be easily and inexpensively deployed in most home and office environments, which makes them more promising.

Due to the impressive ability of deep learning models to capture complex patterns, they have achieved success in many fields. This is meaningful when attempting to utilize deep learning in building a recognition system that maps Wi-Fi signals and gesture actions to each other [14,28,29,30]. When applied to multimedia devices, the machine can identify user gestures through Wi-Fi signals. Although promising, such solutions are based on an assumption: the training and test cases are selected at random and autonomously from a common sample space [14]; accordingly, they only achieve a good performance in a specific domain (in this work, we designate variables that are unrelated to gesture as domain factors, including environments, users, position, direction, and device deployments). Once the domain changes, the assumption breaks and the performance of these systems sharply decreases. The reason for this is that the signals contain significant unfavorable environmental feedback unrelated to hand movements, causing the test distribution to diverge from the training distribution.

A straightforward way to address this domain-dependency challenge is to collect a massive amount of data spanning diverse domains. Unfortunately, this is infeasible in practical scenarios due to difficulties in data collection. Some recent studies have tried to address this issue, but these still have some drawbacks. Firstly, the prevailing approach at present is to employ adversarial techniques [31,32,33] to mitigate the influence of domain-specific variables or utilize transfer learning methods [34,35] to enhance the model’s adaptability to various domains. Both of these approaches can effectively enhance the model’s capacity for cross-domain generalization within a specified range. However, these methods need a large number of measurements to retrain the pre-trained models when facing new target domains, which is in conflict with the needs of real-world scenarios. Second, some existing systems [36,37] rely on multiple transceivers to extract one-size-fits-all features and assume that the locations of the devices are known; however, in in real scenarios, Wi-Fi devices may be deployed in any location. Moreover, all of the aforementioned methods only assume a fixed transceiver deployment. This assumption, however, usually cannot hold, since each environment usually has a personalized device deployment.

**Figure 1:**
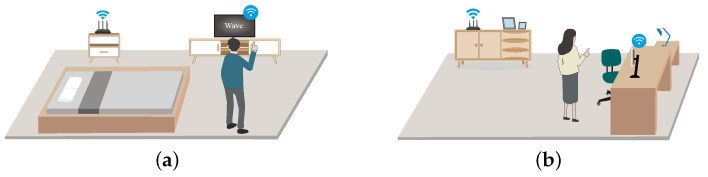
Cross-domain example scenarios, where different users may execute gestures in different settings: (**a**) home; (**b**) office.

To handle the aforementioned limitations, we present Wi-AM, a Wi-Fi-based privacy-preserving gesture recognition system. Leveraging one pair of Wi-Fi transceivers, Wi-AM enables rapid domain adaptation by utilizing just one sample of each gesture from an unseen domain. These advantages significantly reduce the deployment cost and data collection overhead. Furthermore, our experimental results demonstrate that Wi-AM can deliver precise gesture recognition across various domains when confronted with domain changes, such as changes in user location and orientation. With this cross-domain capability, a privacy-preserving gesture recognition system quickly adapts to a new domain, as illustrated in Figure 1.

Practical challenges were faced when attempting to achieve our objective. The primary challenge we confronted is addressing the issue of non-independent identical distribution across domains while safeguarding the unique features of human gestures in Wi-Fi signals from being adversely impacted by different domains. Since the received signals are highly sensitive to the environment during propagation, once one of the domain factors changes, it causes a distribution shift in the received signal pattern, thus affecting gesture recognition. Therefore, it makes sense to devise a methodology to isolate gesture features while mitigating the impact of external factors.

To overcome the initial problem, we adopted a multi-domain adversarial framework composed of three essential components. By maximizing the performance of gesture label prediction while minimizing the performance of domain label prediction, the feature extractor can effectively eliminate the influence of multiple domain factors simultaneously. Note that, different from the traditional single domain adversarial scheme [29], the multiple adversarial domain discriminators include multiple domain discriminators, which can more clearly match the distribution patterns of different domains.

However, our results show that merely using a multi-domain adversarial framework cannot effectively remove the impact induced by the domain factors. Consequently, the second challenge involves adapting the trained model to an unseen domain using limited samples or a single sample. Traditional deep-learning models generally require the use of many samples to avoid overfitting. Nevertheless, this collection process requires extensive data collection and leads to a large training overhead.

To solve this hurdle, we designed an instructional strategy to “teach” the previously mentioned gesture feature extractor model to learn efficiently with small samples while maintaining a good performance and generalization. We first use a task-generation scheme (each task consists of a few samples for each gesture.) to reuse the training data. This approach divides the data from different domain factors into datasets; the data in each unique domain generate a task. All tasks can be used to simulate a large number of domain variations. Then, each task is iteratively fed into the base model to minimize the prediction loss. A major benefit of this method is its capacity to utilize a limited number of samples to facilitate successful model adaptation, thereby enabling a good performance in an unseen domain with minimal samples.

We used a publicly available dataset Widar 3.0 [36] to evaluate Wi-AM’s performance. Wi-AM can deliver an average accuracy of 82.13% and 86.76% for one and three data samples in an unseen domain, respectively. Furthermore, we employed two Wi-Fi public datasets to validate the generalization ability of Wi-AM.

**Contributions:**

- We present Wi-AM, an innovative gesture recognition system that utilizes a pair of Wi-Fi transceivers to achieve an accurate cross-domain performance while requiring only one sample per gesture.
- We design a multi-domain adversarial scheme that aims to eliminate the negative impact of different domain factors on the data distribution while retaining valid information related to gestures. Furthermore, we introduce a new meta-learning framework to implement an updated model of one sample in a new domain for accurate gesture classification.
- Comprehensive evaluations in cross-domain situations demonstrate the effectiveness of Wi-AM.

## 2. Related Work

### 2.1. Wi-Fi-Based Cross-Domain Sensing Technology

By analyzing the transformation of Wi-Fi signals, it is possible to recognize different gestures, which has significant importance in intelligent multimedia technology. However, Wi-Fi wireless signals are immensely responsive to domain-specific changes, including variations regarding the user’s orientation and location. Thus, to tackle the difficulties associated with cross-domain Wi-Fi-based systems, numerous researchers have put forth various solutions. These solutions can generally be separated into two key approaches: transfer learning and domain-independent-based.

Domain-independent-based approaches extract domain-independent gesture features either by building mathematical models or by elaborating deep learning networks. For example, Widar 3.0 [36] proposes a new feature based on Doppler shift called the Body-coordinate Velocity Profile (BVP) by modeling the relationship between gesture activity and channel state information (CSI) signals. The authors developed a universal deep learning model that utilizes domain-independent features extracted from the Body Vibration Pattern (BVP) for gesture recognition. However, calculating the BVP requires the location information of multiple devices and the use of multiple device locations. Some methods employ adversarial networks for domain-independent feature extraction, drawing inspiration from the concept of a Generative Adversarial Network (GAN) [38], and EI [29], which use conditional adversarial architectures to eliminate the influence of environmental factors. JADA [39] uses adversarial networks to map datasets to a domain-agnostic feature representation. However, approaches that rely on adversarial networks often necessitate a substantial amount of data specific to the target domain. Consequently, recent studies have focused on employing sample-generation methods to augment the available dataset. For example, GrossGR [40] uses adversarial networks to generate synthetic training samples, but this still requires a massive amount of training data. In addition, the generative adversarial network suffers from pattern collapse [41].

Transfer-learning-based cross-domain gesture recognition systems [42,43,44] employ fine-tuning schemes to move recognition models to a new domain to avoid re-training models. For instance, CrossSense [43] uses a mixture-of-experts approach to perform transfer learning. However, such solutions still require a large amount of target domain data, and additional costs are still required.

Unlike these methods, the conditions for cross-domain gesture recognition by Wi-AM are even simpler. We only need a small sample of each gesture in the new domain and do not need a large amount of carefully deployed Wi-Fi transceivers as an aid; only a pair of transceivers is required.

### 2.2. Meta-Learning-Based Wi-Fi Sensing Techniques

The main principle of meta-learning is to use prior knowledge and experience to guide the learning of new tasks, enabling models to acquire the ability to learn. This characteristic allows for meta-learning to quickly adjust training parameters for a new learning task, utilizing some of the samples. Therefore, meta-learning is commonly used to address the issue of small-sample problems. Additionally, due to its adaptability to various learning tasks, meta-learning can also be applied to cross-domain problems. In some recent attempts, researchers have utilized meta-learning techniques to address the challenges in deep-learning-based wireless sensing. For example, MatNet-eCSI [45] uses matching networks, a type of meta-learning method for few-shot learning. MatNet-eCSI [45] enables effective gesture recognition using only one sample from each activity in the test environment. RF-Net [46] also utilizes a metric-based meta-learning framework for one-shot gesture recognition. Moreover, RF-Net [46] adopts a more complex feature extraction method. However, these methods focus on one-shot gesture recognition. While these approaches employ carefully designed feature extraction methods to extract gesture information from signals, the authors overlook the strong correlation between Wi-Fi signals and the domain, such as the environment or user. One recent work, OneFi [30] employs a fine-tuning-based one-shot learning framework. This utilizes the powerful feature extraction abilities of the transformer [47] to extract gesture information from Wi-Fi signals. In order to train the OneFi model, the authors designed a virtual gesture generation module to generate virtual samples for data augmentation. However, OneFi [30] requires multiple transceivers, resulting in a substantial installation cost.

In contrast, in this paper, we analyze the correlation between signals and domains. Specifically, we examine the differences in signals obtained by performing different gestures under different domains. Therefore, we utilize adversarial networks to remove domain information from Wi-Fi signals. Subsequently, we employ meta-learning techniques to train a few-shot gesture recognition model, ultimately achieving high-precision cross-domain gesture recognition. This method requires only a limited number of samples from each new domain, making it highly efficient and effective. Moreover, Wi-AM only needs one set of transceivers to achieve accurate gesture recognition across multiple domains. Importantly, Wi-AM does not require intricate expertise and can seamlessly extend to various sensing tasks.

## 3. Background and Motivation

In this section, we first present the Wi-Fi signal transmission model, and we then discuss the motivation behind our research by addressing the challenges associated with cross-domain gesture recognition.

### 3.1. Signal Transmission Model

**Figure 2:**
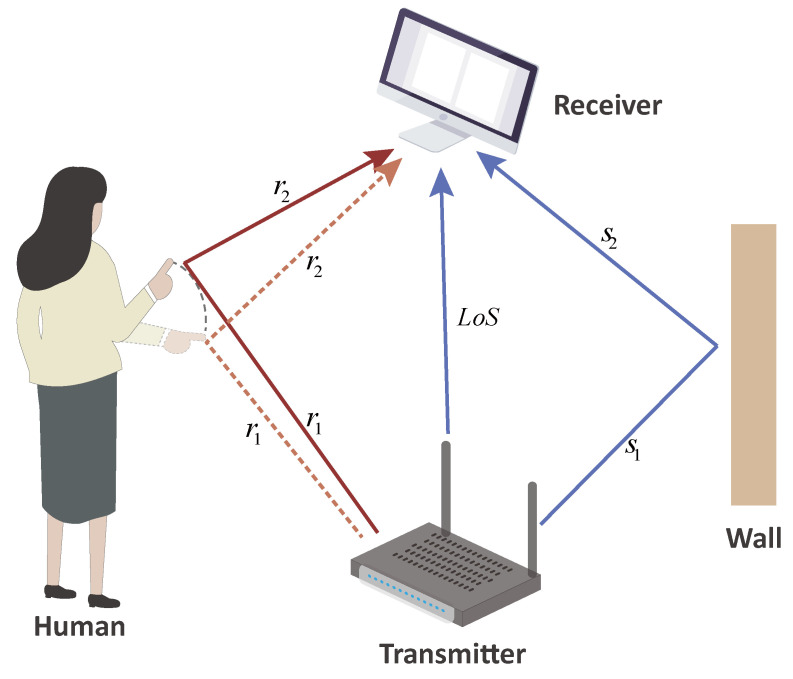
A description of the transmission model of Wi-Fi signals.

Figure 2 describes a typical transmission model of a wireless signal within an indoor setting. When Wi-Fi signals propagate through a wireless channel, this portion of the signals’ propagation path is referred to as the static path [48]. Another portion of the signals will be disturbed by objects and human activities, undergoing multiple reflections and refractions before reaching the receiver, which is known as the dynamic path [48]. In fact, the receiver receives a superposition of signals from these different paths, resulting in a multipath effect. The channel frequency response is presented as follows:

(1) $$\begin{array}{c} H\left(f,t\right)=H_{s}\left(f,t\right)+H_{d}\left(f,t\right)\;=H_{s}\left(f\right)+\sum_{i\in P_{d_{n}}}\alpha_{i}\left(f,t\right)e^{-j2\pi \frac{\tau_{i}\left(t\right)}{\lambda }} \end{array}$$

where $H_{s}\left(f,t\right)$ is the sum of all static paths’ responses. $H_{d}\left(f,t\right)$ denotes the dynamic multipath response that causes a Doppler frequency shift. Specifically, $P_{d_{n}}$ is a representation of the dynamic paths reflected by human bodies. *f* refers to the frequency of subcarriers, *t* denotes the subcarriers’ arrival time, and *N* represents the number of multipaths. According to Equation (1), the length of the dynamic multipath changes when a person executes a gesture. Different gesture actions are capable of causing different length changes in the reflection path, resulting in collected signals that exhibit distinct variations. Thus, we can employ the collected channel state information (CSI) to infer different gestures, meaning that CSI can provide useful information about wireless channel quality and communication link characteristics.

### 3.2. Motivation

#### 3.2.1. Impact of Domain Variation

**Figure 3:**
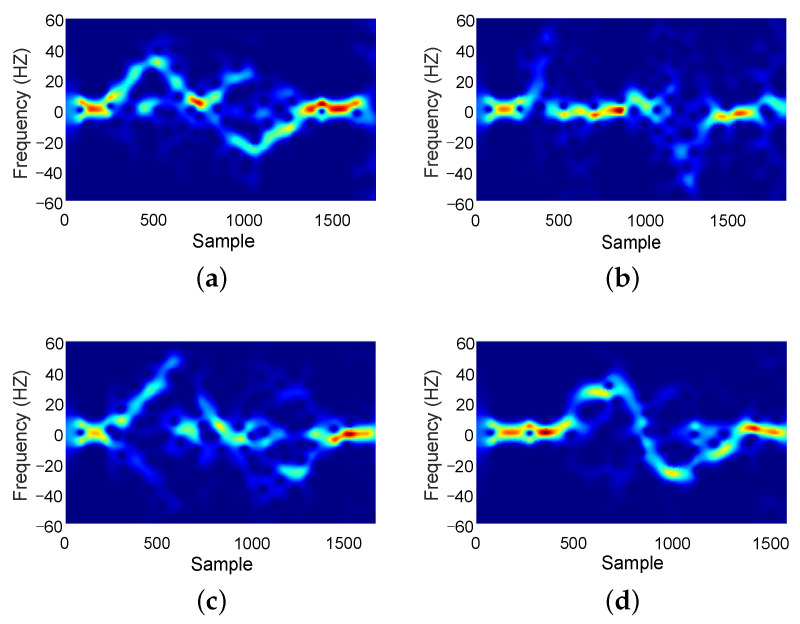
DFS spectrograms under different conditions, as follows: (**a**) User1 performs the “Push&Pull” gesture; (**b**) User1 performs the “Sweep” gesture; (**c**) User1 performs a “Push&Pull” gesture in a different location; (**d**) User 2 performs a “Push&Pull” gesture.

Deep learning methods have proven effective in achieving satisfactory recognition accuracy for Wi-Fi-based gesture recognition systems in specific environments. Indeed, these methods are often based on strong assumptions and heavily rely on numerous environmental factors, including the distribution patterns of features in the environment, the positions of transmitters and receivers, user characteristics, and even user position and direction. These various factors are collectively referred to as the domain [14]. It is crucial to acknowledge that the factors within the domain can introduce unfavorable environmental information, which may be unrelated to the actual gestures being performed, into the collected signals, potentially resulting in performance degradation. To visually demonstrate the impact of various domain factors on the signal, we utilized the experimental data from the Widar 3.0 dataset [36]. Through the visualization of gesture information extracted from the raw signal using the Doppler spectrogram, we showcased how different domain factors influence the signal. First, we selected user 1 to perform two different gestures, “Push&Pull” and “Sweep” gestures, respectively, for which the domain factors do not change. Then, we obtained Doppler frequency shift (DFS) spectrograms by using Short-Term Fourier Transform (STFT) [36]. Figure 3a,b present the results, illustrating that different gesture movements will cause different patterns of Doppler changes. This observation highlights various domain factors that impact the signal’s variation.

To explore how different domains impact gesture recognition performance, we chose different domain factors of location and person, and we ensured the invariance of other domain factors without changes is noteworthy. Figure 3c shows User 1 performing a “Push&Pull” gesture in a different location, while Figure 3d shows User 2 performing a “Push&Pull” gesture. Through our comparative experiments, we discovered that the Doppler spectrogram generated from signals collected under different domain factors for the same gesture action contains distinct gesture information, resulting in a worse gesture recognition performance.

#### 3.2.2. Influence of the Training Data Volume

**Figure 4:**
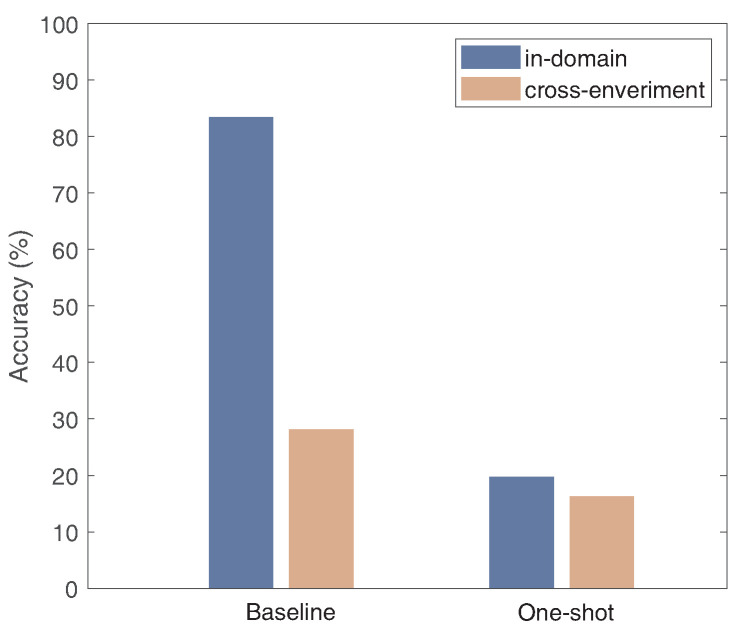
Accuracy of gesture recognition in different environments and with different sample sizes.

One potential way to solve the cross-domain problem presented above is to collect enough training data under different domains. However, this requires extensive human effort. To further investigate the effect of gesture recognition between different domains and sample sizes on gesture recognition performance, we used a network based on SignFi [28] to recognize gestures from the same environment and from different environments. The SignFi network utilizes a nine-layer convolutional neural network(CNN) as a classification algorithm, which is a classical deep learning network for wireless signal classification. As depicted in Figure 4, we discovered that Wi-AM achieves an accuracy of 83.3% in the same environment. However, the accuracy in different experimental environments degrades to 28.1%. Moreover, we found that deep learning methods often require a considerable quantity of data samples to realize optimal performance, and when we reduced the number of gestures used for testing to one, the gesture recognition accuracy was lower than 20% for both intra-domain and cross-domain experiments. These results demonstrate that we need to seek a new method to overcome the above issues.

## 4. System Overview

**Figure 5:**
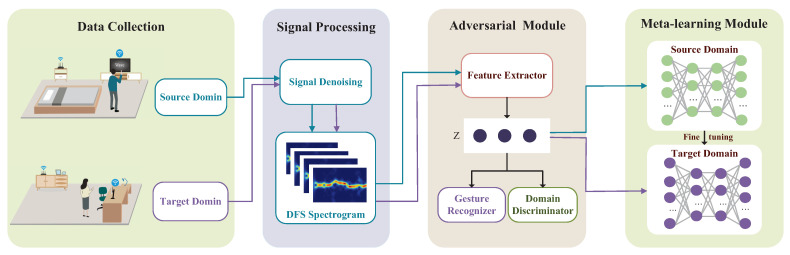
Overview of Wi-AM.

In this paper, our proposed gesture recognition system, named Wi-AM, aims to achieve a high cross-domain recognition performance with minimal human effort, i.e., a small number of target samples. Wi-AM consists of three key components: a preprocessing module, an adversarial domain generalization module, and a cross-domain few-shot gesture meta-learning module. Figure 5 plots the system overview. Our objective in the signal preprocessing module is to extract the Doppler spectrum map from the raw CSI measurements. To remove data noise, we apply a filtering process to the initial CSI signal, followed by a principal component analysis method on the initially denoised signal to extract the significant dynamic components for further denoising, and we finally extract the Doppler spectrum map via STFT. The spectrogram obtained from the CSI signal after the data pre-processing module includes gesture information and gesture-independent domain features, which have a negative effect on our gesture recognition system. We thus designed a domain countermeasure module, which includes three parts: feature extraction, a gesture classifier, and a domain discriminator. This module is used to extract relevant features related to gestures and eliminate domain interference information. The gesture classifier is used to capture gesture-related information. To simultaneously remove multiple domain impacts, we designed multiple-domain discriminators, each of which can focus on the number of data points per domain through the predicted probability distribution obtained by the gesture classifier, which allows the system to achieve a more fine-grained domain adaptation. Finally, we introduce a meta-learning network to fine-tune the parameters to achieve a good cross-domain gesture performance in an unseen domain with one or a few gesture samples.

## 5. System Design

In this section, We first introduce the signal preprocessing module. We then introduce an adversarial domain generalization module. And finally, we present a meta-learning module to enable cross-domain gesture recognition with minimal samples.

### 5.1. Signal Preprocessing Module

We utilized CSI to generate the Doppler offset caused by gestures. The Doppler spectrogram can provide high-precision channel feature information to distinguish different gestures. However, thanks to the inherent hardware imperfections induced by Wi-Fi devices, collected CSI measurements are typically modeled as follows:

(2) $$H\left(f,t\right)=e^{-j2\pi \Delta ft}(H_{s}\left(f\right)+\sum_{i\in P_{d_{n}}}\alpha_{i}\left(f,t\right)e^{-j2\pi f\frac{\tau_{i}\left(t\right)}{\lambda }})$$

where $H_{s}\left(f\right)$ represents the signal collected from all stationary paths of *s*, and $P_{d_{n}}$ represents the count of dynamic paths, where $e^{-j2\pi \Delta ft}$ is the phase offset induced by carrier frequency offsets [14].

**Figure 6:**
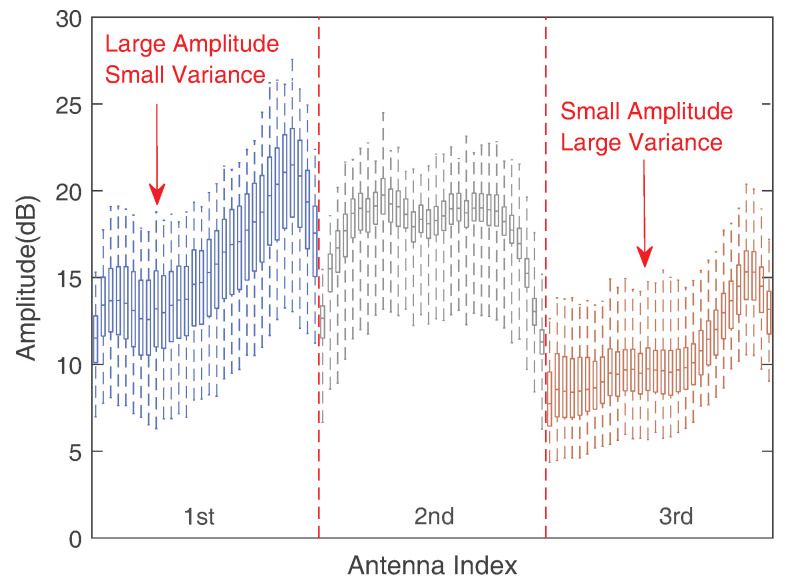
Antenna selection.

These offsets hinder us from obtaining accurate and effective Doppler spectrograms. Fortunately, different antennas of the identical Wi-Fi (NIC) have congruent phase offsets [49]. Hence, we eliminated the phase shift by computing the conjugate multiplication between antennas sharing the same phase offset. For antenna selection, our choice was based on two observations [50]: CSI signals exhibiting greater amplitudes tend to exhibit more pronounced static responses, whereas CSI signals with higher variance tend to show more significant dynamic responses. Figure 6 represents our approach to antenna selection. We can easily observe that the first antenna has the largest amplitude and relatively small variance, and the third antenna has the largest variance and relatively low amplitude. The ratio of amplitude to the CSI signal’s standard deviation serves as an indicator when selecting antennas. Therefore, the first antenna and third antenna have the largest and smallest amplitude and standard deviation scale factors, respectively, and served as our antenna selection.

To eliminate the static component and high-frequency noise, both high-pass and low-pass filters were adopted. We further applied principal component analysis (PCA) to retain the retain the significant components caused by the target movement [48]. Finally, we leveraged STFT to extract the Doppler spectrogram.

### 5.2. Adversarial Domain Generalization Module

**Figure 7:**
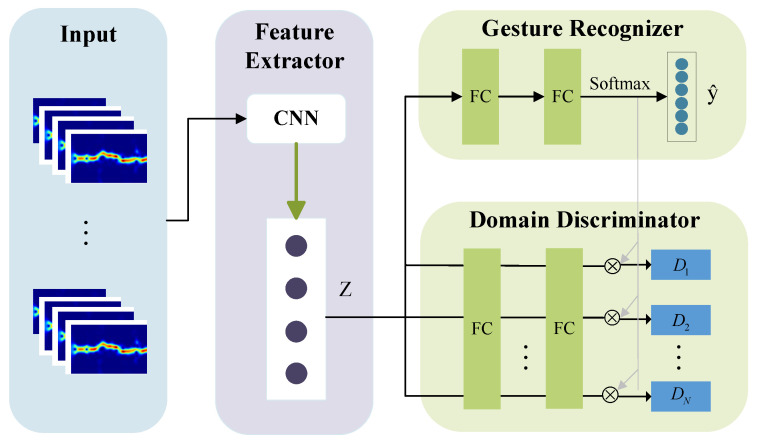
Adversarial domain generalization module.

Due to the significant irrelevant interference introduced by multipath effects in the acquired DFS spectrogram, performance is degraded when transitioning to a new domain. Thus, to further eliminate the influence of different field distribution changes and learn effective features related to human gesture activities, we designed an adversarial domain generalization module, as depicted in Figure 7. This module comprises three main components: a feature extractor block, a gesture classifier block, and a multi-adversarial domain discriminator block. These components work together to achieve the domain-independent extraction of gesture activity information. The feature extractor focuses on extracting the features induced by gestures while eliminating domain information that is unrelated to gestures. The gesture classifier maximizes the accuracy of gesture activity prediction to encourage the feature extractor to learn gesture-related features. Additionally, we developed a multi-adversarial domain discriminator, which can simultaneously remove the impact of multiple domain factors in a multi-domain setting. By maximizing the domain discriminator’s loss, the feature extractor can employ its utmost capabilities to outwit the discriminator through its best effort. Through this minimax game, ultimately, the gesture feature extractor can obtain representations irrespective of domain. Next, we will describe each part in detail.

#### 5.2.1. Feature Extractor

**Figure 8:**
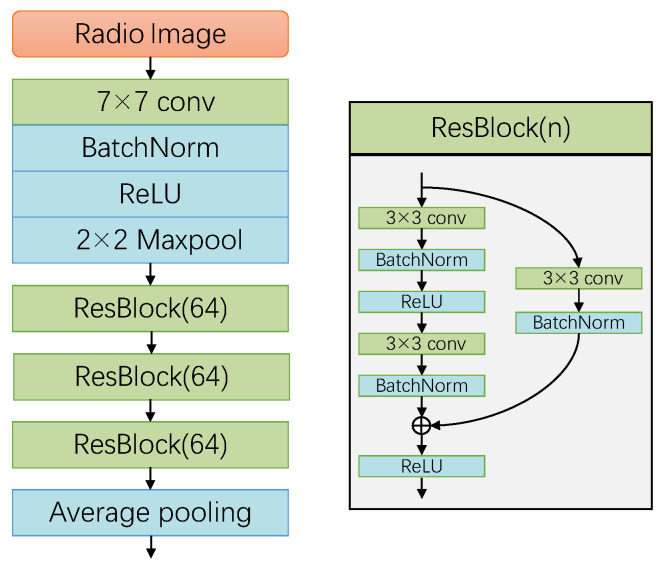
Structure of the feature extractor.

CNN is commonly employed for learning tasks and is capable of extracting relevant and useful features from wireless signals. Therefore, in the gesture feature extraction module, we utilized CNN to extract gesture features. CNN can learn more transferable gesture features by interpreting the factors that change after domain unbinding. Specifically, we selected a residual neural network as a basic feature extractor network [51], as described in Figure 8. The radio image was preprocessed to the same size, serving as input data for the network. The input data were first convolved and then processed through the batch normalization layer [52] and maximum pooling. This was followed by a residual structure with three connected residuals, including the convolutional layer and batch normalization layer [52]. The final layer uses an averaging pooling layer for the output feature representation and the feature encoder’s output features can be expressed as follows:

(3) $$Z=F_{b}\left(X;\theta_{b}\right)=CNN\left(X;\theta_{b}\right)$$

where $\theta_{b}$ represents the parameters of $F_{b}\left(X;\theta_{b}\right)$, such as weights and biases.

#### 5.2.2. Gesture Classifier

The gesture classifier $G_{y}$ is connected to the feature extractor $F_{b}$, and by maximizing the accuracy of recognizing gesture labels, it helps us obtain gesture-related features. After passing through the feature extractor, we obtained all the features containing both gesture-related and other domain-related features. In order to accurately predict gesture labels, we first utilized two fully connected layers to flatten extracted features. Then, we used a softmax layer to map the features and calculate the probability distribution of the predicted labels for the input gesture:

(4) $$\hat{y}=G_{y}\left(F_{b}\left(X;\theta_{b}\right);\theta_{y}\right)$$

The parameters of the gesture classifier, including weights and biases, are represented by $\theta_{y}$ in the equation.

We obtained the predicted probability $\hat{y}$ for each gesture sample through the gesture classifier $G_{y}$. We then computed the loss as follows:

(5) $${\mathrm{Loss}}_{y}=-\frac{1}{\left|X_{s}\right|}\sum_{i=1}^{\left|X_{s}\right|}\sum_{n=1}^{N}y_{in}\log\left(G_{y}\left(F_{b}\left(X_{i};\theta_{b}\right);\theta_{y}\right)\right)$$

where $\left|X_{s}\right|$ and *N* are the number of gesture samples and categories of gesture samples, respectively. We minimized the $Loss_{y}$ of Equation (5). To attain the highest recognition accuracy of the gesture classifier, we trained both the feature extractor and the gesture classifier simultaneously. By simultaneously optimizing these components, we can maximize the performance of the gesture classifier in accurately recognizing different gestures.

#### 5.2.3. Multi-Adversarial Domain Discriminator

The multi-adversarial domain discriminator is designed to minimize domain differences and promote the acquisition of transferable gesture features. Its essence is a form of adversarial learning, similar to the concept of GAN [38]. The feature extractor $F_{b}$ confuses the domain discriminator as much as possible while the domain discriminator tries to distinguish to which domain the sample belongs. Our desired outcome is to obtain domain-invariant features with similar distributions throughout the feature extractor, regardless of domain. We designed a multi-adversarial domain discriminator based on the observation that, in practical domain adaptation problems, users’ gesture activities are often influenced by complex environments. The traditional method [29] just uses a single adversarial network to remove the impact of a single domain. However, when faced with more complex domain data distributions, such methods often fail to match different domain distribution patterns to the maximum extent. This limitation causes the feature extractor to not extract clean gesture features. Our proposed multi-adversarial domain discriminator addresses this issue by using multiple discriminators to better match the distributions between domains and thus extract more domain-invariant gesture features.

Therefore, the multi-adversarial domain discriminator we designed consists of K domain discriminators $G_{d}^{k}$, each associated with one of the K gesture categories. Then, we adopted an attention mechanism for use in the discriminators’ block by weighting their features $F_{b}\left(x_{i}\right)$ with the probability ${\hat{y}}_{i}^{k}$. Thus, we could obtain the predicted labels ${\hat{d}}^{k}$ for the gesture feature domain from each domain discriminator:

(6) $${\hat{d}}^{k}=G_{d}^{k}\left({\hat{y}}^{k}F_{b}\left(X,\theta_{b}\right);\theta_{d}^{k}\right)$$

where $\theta_{d}^{k}$ represents the k-th discriminator’s parameters. Training multi-domain discriminators with the probability-weighted data point ${\hat{y}}^{k}F_{b}\left(X,\theta_{b}\right)$ allows each domain discriminator to focus more on the relevant data points. By avoiding the forced alignment of distinct distribution patterns, our approach effectively mitigates negative transfer. Simultaneously, positive transfer is enhanced for each instance through the utilization of domain discriminators with varying parameters. We calculated the loss of each domain discriminator by using the domain prediction label distribution obtained through each domain discriminator $G_{d}^{k},k=1,2,\cdots ,K$. Then, the total loss of domain discriminators $G_{d}$ is expressed as follows:

(7) $${\mathrm{Loss}}_{d}=-\frac{1}{\left|X_{s}\right|}\sum_{k=1}^{K}\sum_{i=1}^{\left|X_{s}\right|}\sum_{n=1}^{N^{k}}d_{in}^{k}\log G_{d}^{k}\left({\hat{y}}^{k}F_{b}\left(X_{i},\theta_{b}\right);\theta_{d}^{k}\right)$$

where $\left|X_{s}\right|$ and $N^{k}$ represent the number of gesture samples and domains. $d_{in}^{k}$ represents the domain label.

Then, according to Equations (5) and (7), the final loss can be expressed as follows:

(8) $$Loss={\mathrm{Loss}}_{y}-\lambda {\mathrm{Loss}}_{d}$$

where λ is the weighting parameters. Through minimizing the final loss function Loss, we can minimize $Loss_{y}$ while maximizing $Loss_{d}$, making the domain discriminator unable to identify domain information, and finally obtain domain-independent gesture feature information.

Unfortunately, although the adversarial domain generalization module can eliminate part of the domain information, it is still hard to remove all impacts induced by complex domain factors.

**Figure 9:**
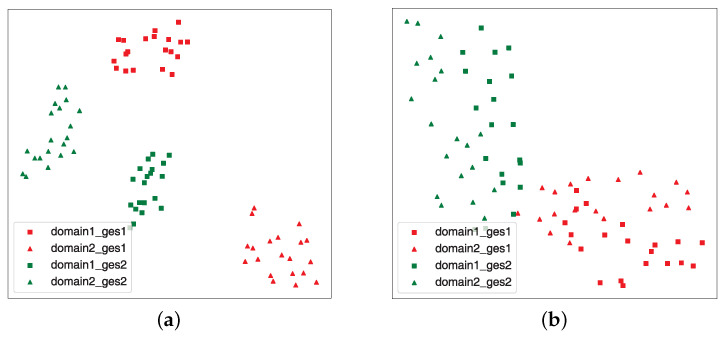
t-SNE visualization: (**a**) The initial distribution; (**b**) Distribution of features extracted by adversarial processing.

To visualize the above phenomenon, we used t-SNE visualization [53] to downscale the feature to two dimensions to show the feature distribution processed by the adversarial domain generalization module. As plotted in Figure 9, we selected two different users as two separate domains, in which different gestures are executed. Figure 9a shows that the boundaries of the four distributions are clear for the different gestures in the two different domains. In theory, after the original sample has been processed by the adversarial domain generalization module, the extracted features should contain human gesture information while the domain information is eliminated. Figure 9b shows the distribution of features extracted through the adversarial process. Compared to the initial data distribution, the processed feature distribution has clear boundaries between the different samples, with no overlap at all, while for the different domains, the sample distributions are able to cluster successfully, but we can observe that the clusters do not completely overlap and do not arrive at a true complete separation of the domain features. Thus, it is desirable to seek a new method to achieve an accurate cross-domain performance.

### 5.3. Meta-Learning Training Module

As described above, merely using the proposed multi-domain adversarial scheme can only partially eliminate the influence of the domain, which cannot ensure good cross-domain gesture recognition. To overcome this obstacle, we adopted a meta-learning scheme. The basic idea is to construct learning tasks during training, representing different domains, and use them to train a mature model that provides good initialization and can adapt quickly to new tasks. This scheme allows us to achieve accurate gesture recognition in new domains with a few samples.

**Figure 10:**
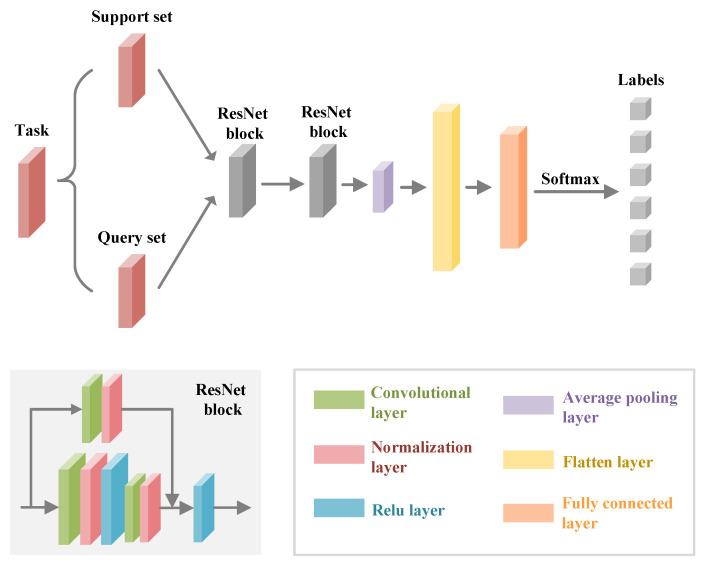
Structure of the base network.

Figure 10 illustrates the structure of the base network. Specifically, we utilized a backbone network containing two resnet blocks, which has four convolutional layers. This can be used to better learn the high-level features of gestures. After that, the features were passed through a fully connected layer and classified using a softmax layer. Finally, we obtained the predicted labels. We divided the classification learning model network into a feature extractor block $f_{\theta }^{e}$ and a gesture classifier block $f_{\theta }^{c}$, where ${\left\{\omega_{n}\right\}}_{n=1}^{N}$ represents the weight vector of the classification head. Traditional meta-learning frameworks, such as MAML [54], initialize all parameters randomly, resulting in unwanted disturbances [55]. To enhance the categorization efficacy of meta-learning in the presence of novel categories, we adopted an improved meta-learning approach, which focuses on initializing the classification layer using prototypes derived from training data. Specifically, we employed a prototype network to bolster the meta-learning classifier [56]. The class prototype $\epsilon_{n}$ was calculated by taking the mean of the embeddings created by the feature extractor $f_{\theta }^{e}$ for each training example of the class. The class prototype $\epsilon_{n}$ obtained through computation was used to initialize the parameters ${\left\{\omega_{n}\right\}}_{n=1}^{N}$ of the classification layer $f_{\theta }^{c}$. This approach leads to a faster convergence compared to using random parameter initialization. Such an initialization reduces unnecessary fluctuations during model training, helping the model adapt to new tasks more rapidly, and thereby improving training speed.

The execution stage of the meta-learning module was divided into two phases: the meta-training phase $S_{train}$ and the meta-testing phase $S_{test}$. During the meta-training phase, we constructed a training dataset using extensive gesture samples from the source domain to initialize a well-trained meta-learning model. In the meta-testing phase, we utilized a small number of measurements from the target domain to quickly adapt the pre-trained meta-learning parameters. Unfortunately, since the data used in the meta-training and meta-testing phases are not from the same domain, this well-trained initialized meta-learning model could achieve the expected recognition accuracy in the training phase in new domains. Therefore, we propose a domain generalization task generation scheme to augment the model’s generalization capability.

Next, we adopted a task generation method to generate diverse tasks on the training dataset. We utilized the Widar 3.0 public dataset [36], which contains multiple domain factors, including users, locations, and orientations. These factors can combine to form a distinct domain in relation to one another. Specifically, it is assumed that the dataset collects data from *m* different users (*U*), *n* different locations (*L*), and *k* different orientations (*O*); then, different domains within each factor are combined with domains within the other factors. Therefore, our domain range was $U_{1}L_{1}O_{1}-U_{m}L_{n}O_{k}$. Each domain is unique, with each task corresponding to a single domain, and the data within each domain are separate from the others. The benefit of this approach is that the meta-learning model can acquire knowledge of diverse complex tasks through training. As a result, when faced with a new task, the meta-learning model can quickly adapt to it, ultimately achieving cross-domain capabilities. In particular, we utilized the prototype network to optimize the initial classifier of meta-learning [56]. This approach can accelerate the training of the meta-learning model.

Then, each task $T_{i}$ was categorized into a support set and a query set, denoted as $T_{i}=\left(T_{i}^{S},T_{i}^{Q}\right)$. Note that there are no overlapping samples for the two sets, and each class contains only N samples. During the meta-training phase, the model comprised an inner loop and an outer loop. We first extracted a batch of tasks from the training set $S_{train}$ in the inner loop, with each task having corresponding parameters $\theta_{T_{i}}$. Specifically, for task $T_{i}$, we employed cross-entropy ${\mathrm{Loss}}_{T_{i}^{S}}$ to compute the loss of support set $T_{i}^{S}$ and update the parameters $\theta_{T_{i}}$:

(9) $$\theta_{T_{i}}=\theta -\alpha \nabla_{\theta }{\mathrm{Loss}}_{T_{i}^{S}}\left(\theta \right)$$

where α denotes the learning rate of the inner loop. In the outer loop, the model fine-tunes the parameters of $T_{i}^{Q}$ based on the summarized loss obtained from the inner loop training. Then, via backpropagation, the parameters θ are expressed as follows:

(10) $$\theta^{\prime }=\theta -\beta \nabla_{\theta }\sum_{i=1}^{M}{\mathrm{Loss}}_{T_{i}^{Q}}\left(\theta_{T_{i}}\right)$$

where β denotes the learning rate of the outer loop. However, we found that computing two layers of gradients leads to a huge computational burden. Therefore, to tackle this problem, we adopted a parameter approximation method [57] to alleviate the computational burden of MAML [54]. The key idea is to ignore the second-order derivative term. By leveraging a fixed value as the gradient to directly calculate the parameter update, the updated model parameters θ can be presented as follows:

(11) $$\theta^{\prime }=\theta -\beta \nabla_{\theta^{\prime }}\sum_{i=1}^{M}{\mathrm{Loss}}_{T_{i}^{Q}}\left(\theta_{T_{i}}\right)$$

We selected the Adam optimizer [58] to optimize the parameters of the basic model. Through our domain generalization task generation scheme, we generated a diverse set of tasks encompassing various domain variations. This provides the model with good domain generalization adaptability. Algorithm 1 describes the detailed step-by-step algorithm.
**Algorithm 1** Proposed meta-learning recognition module**Input:** Training and testing dataset *S*, inner step size α,meta update step size β**Output:** Few-shot gesture classification model**While** not done **do**    Task generation $T_{i}=\left(T_{i}^{S},T_{i}^{Q}\right)$ from *S*    Compute prototype $\epsilon_{n}$    Generate classifier initialization $\omega_{n}$    Set $\theta =\left\{\omega_{n},\theta_{g(x)}\right\}$    **for** $T_{i}\in T$ **do**        Calculate loss ${\mathrm{Loss}}_{T_{i}^{S}}$        Update the parameter $\theta_{T_{i}}=\theta -\alpha \nabla_{\theta }{\mathrm{Loss}}_{T_{i}^{S}}\left(\theta \right)$    **end**    Update $\theta^{\prime }=\theta -\beta \nabla_{\theta^{\prime }}\sum_{i=1}^{M}{\mathrm{Loss}}_{T_{i}^{Q}}\left(\theta_{T_{i}}\right)$  **end**

## 6. Experimental Evaluations

### 6.1. Experiment Setup

**Figure 11:**
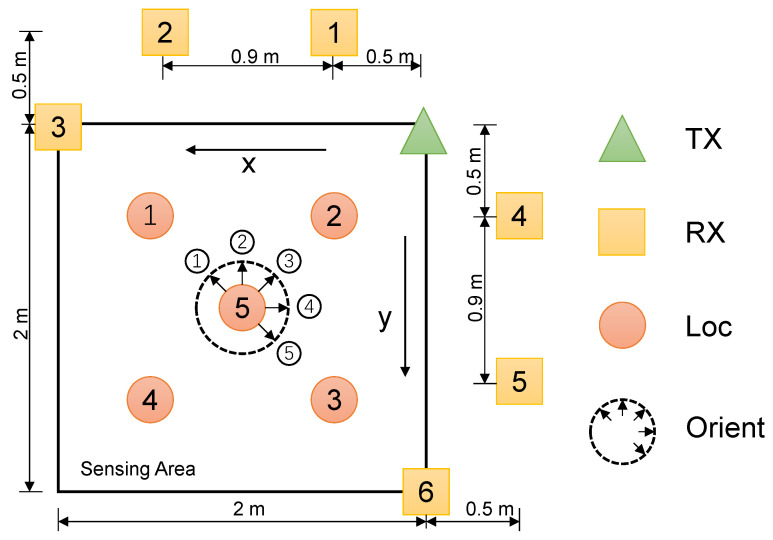
Distribution of equipment in the sensing area; figure modified from Widar 3.0 [36].

We utilized the CSI-based public gesture hand dataset Widar 3.0 [36] for a performance evaluation. We selected data from Widar 3.0 for our experimental evaluation dataset, with details described in Table 1. We used a total of 10 volunteers to perform 6 different gestures in 5 different locations, and 5 different orientations across 3 different contexts. The sensing area was distributed as shown in Figure 11. The Widar 3.0 [36] dataset’s rich variety of domains provided training and testing for our model. For a user performing a gesture in a room, The Widar 3.0 provides different domain factors for 5 locations, 5 orientations, and 6 Wi-Fi transceiver deployments, which can be composed into 150 different domain factors, and there were only 5 samples per gesture in most of the domains. Thus this dataset meets the needs of our experiments, as it contains a cross-domain and uses a small sample size for new domain-adapted gesture recognition.

### 6.2. Overall Performance

**Figure 12:**
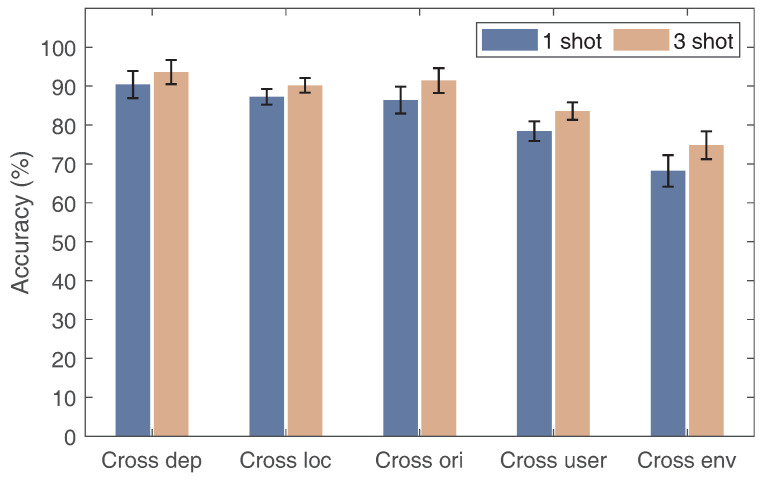
Gesture recognition accuracy on the Widar 3.0 dataset.

Initially, we assessed the system’s performance in cross-domain gesture recognition considering various factors across diverse domains, including the transceiver’s deployment, location, orientation, person, and environment. Specifically, we chose one domain as the target domain to test the cross-domain performance, while the data in other domains were employed for training. Note that when selecting a domain for evaluations of cross-domain performance experiments, other domain factors remain unchanged.The results are plotted in Figure 12, where we can see that Wi-AM’s gesture recognition accuracy ranges from 68.2% to 90.4% for a sample with different cross-domain experiments. Specifically, in the scenario where only one sample is available, the accuracy of gesture recognition is 90.4%, 87.25%, and 86.4% for cross-transceiver deployment, cross-loc, and cross-ori. For the cross-per and cross-env experiments, the accuracy is 78.42% and 68.2%, respectively. This means that the environment and person domain domain variables play a stronger crucial role in the feature distribution. When increasing the number to three gesture samples, we observe that the recognition accuracy surpasses 90%, and cross-direction and cross-transceiver deployments, and increases to 83.6% and 74.8% for cross-user and cross-environment in each case. Overall, the gesture recognition system designed in this paper has excellent application prospects.

### 6.3. Impact of Adversarial Domain Generalization Module

**Figure 13:**
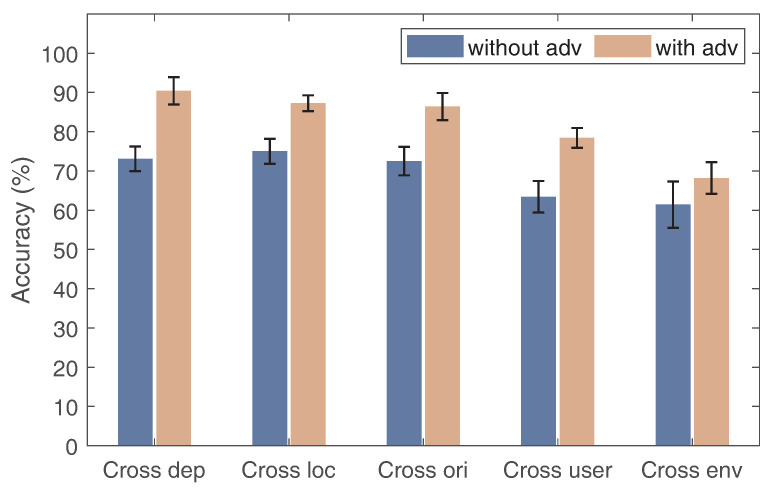
The performance with multi-domain adversarial scheme for each domain factor.

We now evaluate the effect of the proposed adversarial model. Specifically, we remove the domain adversarial module and do not remove the domain adversarial module in the same experimental setup and perform one gesture recognition. In Figure 13, the features extracted by the domain adversarial module are shown to have better recognition accuracy under the classification of the meta-learning scheme. This is because the CSI signal without domain adversarial processing contains interference information related to the environment, while the domain adversarial module can eliminate part of the influence of domain information, thus enhancing the accuracy of primary gesture recognition.

### 6.4. Impact of Meta-Learning Methodology

**Figure 14:**
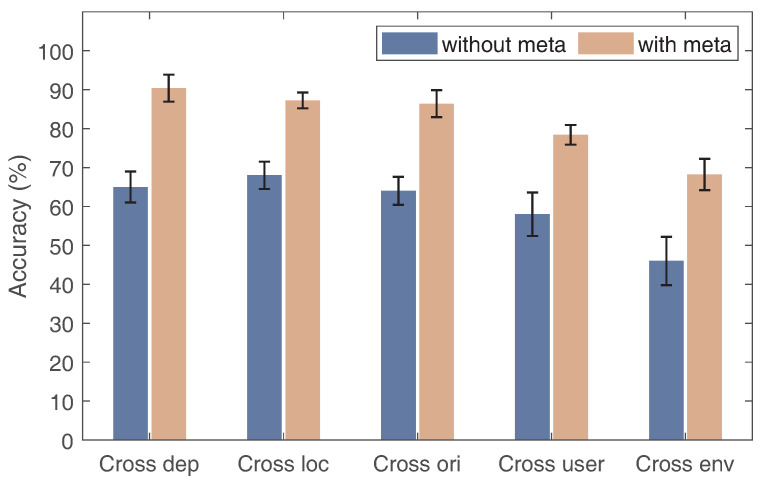
The performance with meta-learning scheme for each domain factor.

To validate the effectiveness of the meta-learning methodology, we conducted an experiment to serve as a baseline for comparison with Wi-AM. In the baseline experiment, we did not utilize meta-learning training strategies. Instead, we solely trained the data using a domain adversarial module. In this experiment, the training data consisted of all source data and one gesture sample from the test domain. Then, we made gesture classification predictions by utilizing the measurements from the test domain. Wi-AM trained a meta-learning model after undergoing domain adversarial processing. The meta-learning strategy simulated a large number of domain variations within the datasets. When facing an unknown target domain, it enabled an accurate gesture recognition performance by adapting to the unseen domain with a single gesture sample. Figure 14 depicts the corresponding results.The meta-learning strategy consistently demonstrates a superior performance compared to the method without the strategy for small sample gesture recognition across domains. The meta-learning strategy learns gesture features from the training data and swiftly adapts within the target domain, underscoring its effectiveness.

### 6.5. Comparison with Other Models

**Figure 15:**
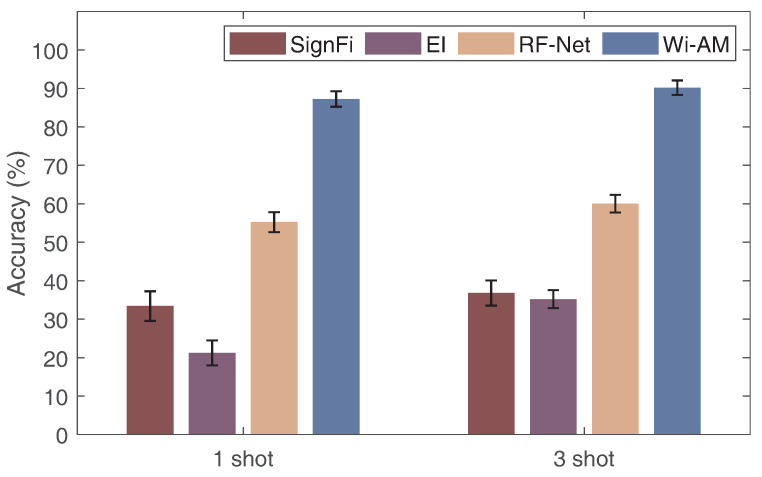
Comparison of recognition models.

To showcase the benefits of Wi-AM, we selected three common gesture recognition methods to perform the comparison, including SignFi [28], EI [29], and RF-Net [46], where SignFi uses a traditional CNN structure, EI uses an adversarial network approach, and RF-Net employs a meta-learning model. Then, we evaluated the cross-location gesture recognition performance in one-shot and three-shot conditions. Figure 15 shows the results. The accuracy is 33.41%, 36.78% and 21.23%, 35.21% for SignFi and EI, respectively. For RF-Net, the accuracy is 55.23% and 64.02% in 1-shot and 3-shot cases, respectively. The recognition accuracy of Wi-AM is 87.25% and 90.2%, outperforming the other three methods. Note that, for the sake of fairness, we added one and three target samples to the training dataset for SignFi to train the model for two cases. Although EI and RF-Net have a cross-domain ability, when only limited target samples are available, these two methods are still not good enough for gesture recognition, but Wi-AM uses adversarial networks and meta-learning to achieve precise gesture recognition. These results demonstrate the superiority of Wi-AM.

### 6.6. Impact of Training Dataset Diversity

**Figure 16:**
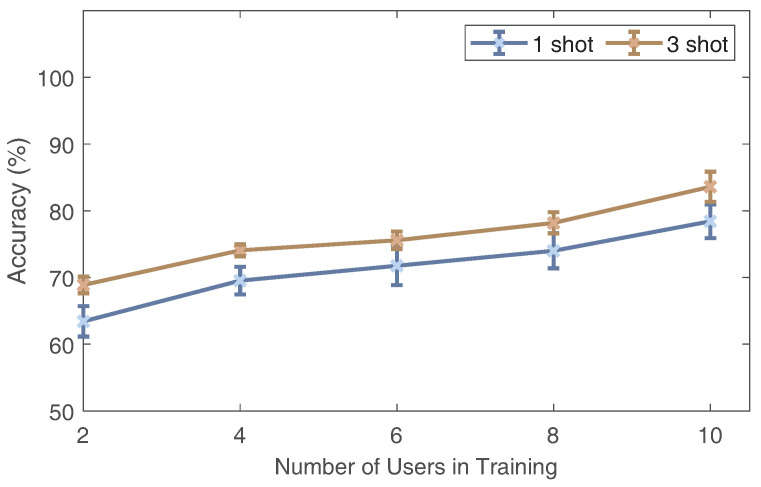
The impact of training dataset diversity.

We varied the number of trained users to explore the implications for cross-domain gesture recognition. We adjusted the number of user-executed gesture datasets used for training from two to ten with two steps. The results are plotted in Figure 16. As the number of trained users increases, the accuracy increases. The accuracy increases to 78.42% and 83.6% for one and three sample settings when using ten users’ data to train the model. Thus, we can safely reach the conclusion that increasing the domain diversity of the training dataset can help Wi-AM learn more and adapt to new domains.

### 6.7. Multiple Datasets to Validate Model Generalization Performance

**Figure 17:**
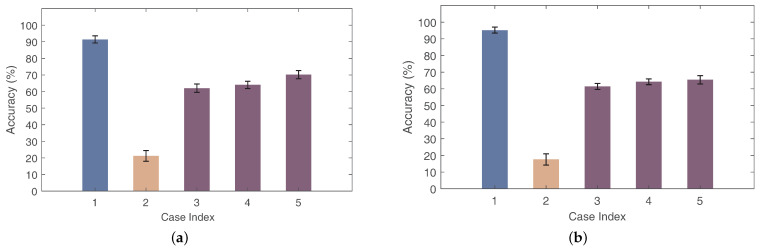
The performance on two public Wi-Fi sensing datasets: (**a**) SignFi Dataset, (**b**) WiAR Dataset.

We chose two other public Wi-Fi datasets to validate the generalization capability of Wi-AM: the first dataset is SignFi [28], which focuses on human sign language gestures, and the measurements were collected from two environments, i.e., lab, and home. In this dataset, we selected 150 sign language gestures from the lab environment, with 7500 instances, performed by five different users, and 150 identical sign language gestures from the home environment, with 1500 instances performed by one user. We used this dataset to explore the cross-environment problem. Specifically, we used the data of five different people in the lab setting as the source to train the model, while we used the data in the home setting as test samples. For the second dataset, WiAR [59] was used. It contains sixteen activities, including coarse-grained activities and gestures, performed by ten volunteers, with each volunteer perfoming an activity 30 times. We used this dataset to explore the cross-user problem, where we randomly used one user as the test domain and the remaining users as the training domains. For two different sets of experiments, we classified five different sets of cases as comparisons. Firstly, for case 1, we used 80% of the total data to train the model and the remaining 20% to test the model. The training method uses the CNN structure in SignFi [28]. For case 2, we directly used the samples in the target domain to test the training model. For cases 3, 4, and 5, we tested the cross-domain performance by using 1, 3, and 5 samples in the target domain for model fine-turning, respectively. In Figure 17, we can see that case 1 has good accuracy, while the gesture recognition accuracy drops sharply in Case 2. This is due to the limited cross-domain capabilities of traditional CNN when it comes to domain changes, while with Wi-AM, the gesture recognition accuracy increases by 40.8% and 43.8%, respectively, compared to case 2 in two different datasets. When using more samples in the target domain, Wi-AM can achieve higher gesture recognition accuracy. Therefore, these results illustrate that Wi-AM has a good generalization capability.

### 6.8. Impact of Crossing Multiple Target Domains

**Figure 18:**
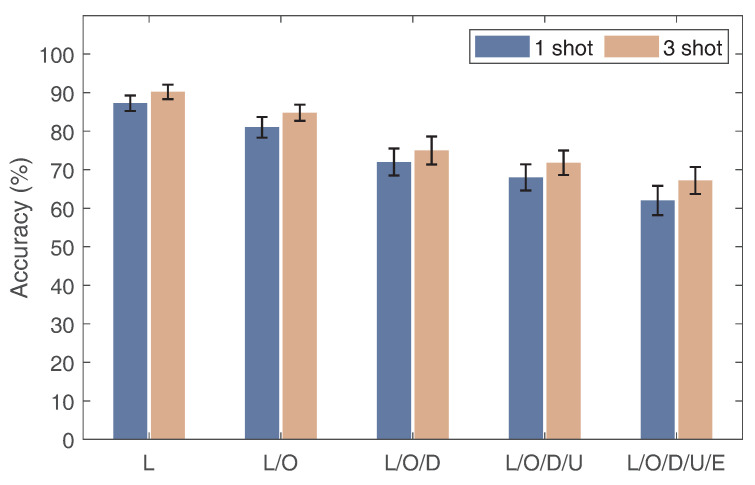
Impact of crossing multiple target domains.

In our previous experiments, we only considered a single domain factor for cross-domain experiments, and the other domain factors were unchanged. In practice, however, there may be multiple domain variations, so, in this experiment, we examined the Wi-AM performance by gradually increasing the number of domain factors. Specifically, we used the Widar 3.0 public dataset, which includes five different domain factors, namely the location, orientation, transceiver deployment, user, and environment. For simplicity, we denoted these five domain factors as L, O, E, U, and D. We selected the domain factor of location (L) as the benchmark and continued to add other domain factors, i.e., L, L/O, L/O/D, L/O/D/U, and L/O/D/U/E. Figure 18 illustrates the results. Wi-AM delivers a one-shot accuracy of 87.25%, 81%, 72.2%, 68%, and 62.2%, respectively. This is because, as the domain factor increases, there is more interference with the gesture information in the acquired signal. Meanwhile, the experimental results show that Wi-AM can be supported across multiple target domains via the addition of more gesture samples.

## 7. Discussion

In the current version, Wi-AM relies on the training dataset containing massive amounts of data from different domains to achieve a good cross-domain performance. By incorporating diverse domain data during training, Wi-AM can significantly enhance its generalization capability. However, Wi-AM still has some limitations. For instance, it can only perform gesture recognition in static environments. When there are other moving objects in the environment, it may introduce interference and increase the difficulty of gesture recognition. Therefore, we need to design more effective signal separation methods for the signal preprocessing stage in our future work. Moreover, multi-user gesture recognition poses a challenging task. When multiple users perform actions, more complex signal mixtures are generate, which the methods used in Wi-AM may not adequately address. We will investigate how to recognize gestures from multiple individuals in our future work. Finally, due to the current implementation, which solely relies on training the model with data from an open dataset, Wi-AM’s generalization ability to unseen domains is somewhat limited. By leveraging additional standard Wi-Fi gesture datasets, we anticipate that Wi-AM can significantly improve its cross-domain gesture recognition abilities, and we also believe that when more and more manufacturers and academic researchers focus on the wireless sensing territory, there will be a larger number of public Wi-Fi public gesture datasets, furthering the actual implementation of the wireless sensing technology in our daily lives.

## 8. Conclusions

This paper introduces Wi-AM, a cross-domain gesture recognition system that uses only a few samples in unseen domains. By designing a multi-domain adversarial network, Wi-AM can eliminate the influence of different domains as much as possible. Then, followed by an improved meta-learning framework, Wi-AM demonstrates remarkable potential for fast domain adaptation, successfully adapting to unseen domains with just a single sample. Extensive field tests demonstrate the feasibility and effectiveness of Wi-AM.

## Figures and Tables

**Table 1:** Description of our evaluation dataset.

Dataset	Environments	Gestures	No. of Users	No. of Locations	No. of Orientations	No. of Transceiver Deployments
Widar3.0	1: Classroom; 2: Hall; 3: Office;	1: Push&Pull; 2: Sweep; 3: Clap; 4: Slide; 5: Draw-O; 6: Draw-Zigzag;	10	5	5	6

## Data Availability

We used three datasets in the paper. These datasets are: Widar3.0 (http://tns.thss.tsinghua.edu.cn/widar3.0/, accessed on 10 December 2023), SignFi (https://yongsen.github.io/SignFi/, accessed on 10 December 2023), and WiAR (https://github.com/linteresa/WiAR, accessed on 10 December 2023). All of these datasets are publicly accessible.

## References

[1] Zhao H., Wang S., Zhou G., Zhang D. (2019). Ultigesture: A wristband-based platform for continuous gesture control in healthcare. Smart Health.

[2] Yu Y., Wang D., Zhao R., Zhang Q. Rfid based real-time recognition of ongoing gesture with adversarial learning. Proceedings of the 17th Conference on Embedded Networked Sensor Systems.

[3] Zou H., Zhou Y., Yang J., Jiang H., Xie L., Spanos C.J. Wifi-enabled device-free gesture recognition for smart home automation. Proceedings of the 2018 IEEE 14th International Conference on Control and Automation (ICCA).

[4] Kim M., Cho J., Lee S., Jung Y. (2019). Imu sensor-based hand gesture recognition for human-machine interfaces. Sensors.

[5] Sbernini L., Quitadamo L.R., Riillo F., Lorenzo N.D., Gaspari A.L., Saggio G. (2018). Sensory-glove-based open surgery skill evaluation. IEEE Trans. Hum.-Mach. Syst..

[6] Zhang Y., Chen Y., Yu H., Yang X., Lu W., Liu H. (2018). Wearing-independent hand gesture recognition method based on emg armband. Pers. Ubiquitous Comput..

[7] Wang M., Ni B., Yang X. Recurrent modeling of interaction context for collective activity recognition. Proceedings of the IEEE Conference on Computer Vision and Pattern Recognition.

[8] Oyedotun O.K., Khashman A. (2017). Deep learning in vision-based static hand gesture recognition. Neural Comput. Appl..

[9] Despinoy F., Bouget D., Forestier G., Penet C., Zemiti N., Poignet J. (2015). Unsupervised trajectory segmentation for surgical gesture recognition in robotic training. IEEE Trans. Biomed. Eng..

[10] Xue J., Zhang J., Gao Z., Xiao W. (2023). Enhanced wifi csi fingerprints for device-free localization with deep learning representations. IEEE Sens. J..

[11] Gu Y., Yan H., Zhang X., Wang Y., Huang J., Ji Y., Ren F. (2023). Attention-based gesture recognition using commodity wifi devices. IEEE Sens. J..

[12] Yang B., Wang H., Hu L., Zhu H., Lam C.-T., Fang K. (2023). Few-shot cross-domain-based wifi sensing system for online learning in iot. IEEE Sens. J..

[13] Zhang L., Zhang Y., Zheng X. (2020). Wisign: Ubiquitous american sign language recognition using commercial wi-fi devices. ACM Trans. Intell. Syst. Technol. TIST.

[14] Feng C., Wang N., Jiang Y., Zheng X., Li K., Wang Z., Chen X. (2022). Wi-learner: Towards one-shot learning for cross-domain wi-fi based gesture recognition. Proc. ACM Interact. Mob. Wearable Ubiquitous Technol..

[15] He Y., Chen Y., Hu Y., Zeng B. (2020). Wifi vision: Sensing, recognition, and detection with commodity mimo-ofdm wifi. IEEE Internet Things J..

[16] Zou H., Yang J., Zhou Y., Spanos C.J. Joint adversarial domain adaptation for resilient wifi-enabled device-free gesture recognition. Proceedings of the 2018 17th IEEE International Conference on Machine Learning and Applications (ICMLA).

[17] Niu K., Zhang F., Wang X., Lv Q., Luo H., Zhang D. (2021). Understanding wifi signal frequency features for position-independent gesture sensing. IEEE Trans. Mob. Comput..

[18] Gao R., Li W., Xie Y., Yi E., Wang L., Wu D., Zhang D. (2022). Towards robust gesture recognition by characterizing the sensing quality of wifi signals. Proc. ACM Interact. Mob. Wearable Ubiquitous Technol..

[19] Zou Y., Xiao J., Han J., Wu K., Li Y., Ni L.M. (2016). Grfid: A device-free rfid-based gesture recognition system. IEEE Trans. Mob. Comput..

[20] Wang C., Liu J., Chen Y., Liu H., Xie L., Wang W., He B., Lu S. Multi-touch in the air: Device-free finger tracking and gesture recognition via cots rfid. Proceedings of the IEEE INFOCOM 2018—IEEE Conference on Computer Communications.

[21] Zhao R., Zhang Q., Cao D., Sheng Z., Wang D. (2021). Gesture recognition with rfid: An experimental study. CCF Trans. Pervasive Comput. Interact..

[22] Shin D., Yoon J. Multi-point gesture recognition leveraging acoustic signals and cnn. Proceedings of the 2020 International Conference on Information and Communication Technology Convergence (ICTC).

[23] Siddiqui N., Chan R.H. (2020). Hand gesture recognition using multiple acoustic measurements at wrist. IEEE Trans. Hum.-Mach. Syst..

[24] Nouman M., Khoo S.Y., Mahmud M.A., Kouzani A.Z. (2022). Recent advances in contactless sensing technologies for mental health monitoring. IEEE Internet Things J..

[25] Liu H., Wang Y., Zhou A., He H., Wang W., Pan P., Wang K., Lu Y., Liu L., Ma H. (2020). Real-time arm gesture recognition in smart home scenarios via millimeter wave sensing. Proc. ACM Interact. Mob. Wearable Ubiquitous Technol..

[26] Ninos A., Hasch J., Zwick T. Multi-user macro gesture recognition using mmwave technology. Proceedings of the 2021 18th European Radar Conference (EuRAD).

[27] Yan B., Wang P., Du L., Chen X., Fang Z., Wu Y. (2023). mmgesture: Semi-supervised gesture recognition system using mmwave radar. Expert Syst. Appl..

[28] Ma Y., Zhou G., Wang S., Zhao H., Jung W. (2018). Signfi: Sign language recognition using wifi. Proc. ACM Interact. Mob. Wearable Ubiquitous Technol..

[29] Jiang W., Miao C., Ma F., Yao S., Wang Y., Yuan Y., Xue H., Song C., Ma X., Koutsonikolas D. Towards environment independent device free human activity recognition. Proceedings of the 24th Annual International Conference on Mobile Computing and Networking.

[30] Xiao R., Liu J., Han J., Ren K. Onefi: One-shot recognition for unseen gesture via cots wifi. Proceedings of the 19th ACM Conference on Embedded Networked Sensor Systems.

[31] Shi C., Liu J., Borodinov N., Leao B., Chen Y. Towards environment-independent behavior-based user authentication using wifi. Proceedings of the 2020 IEEE 17th International Conference on Mobile Ad Hoc and Sensor Systems (MASS).

[32] Dian C., Wang D., Zhang Q., Zhao R., Yu Y. (2020). Towards domain-independent complex and fine-grained gesture recognition with rfid. Proc. ACM Hum.-Comput. Interact..

[33] Wang J., Wang C., Yin D., Gao Q., Liu X., Pan M. (2021). Cross-scenario device-free gesture recognition based on self-adaptive adversarial learning. IEEE Internet Things J..

[34] Lin C., Hu J., Sun Y., Ma F., Wang L., Wu G. Wiau: An accurate device-free authentication system with resnet. Proceedings of the 2018 15th Annual IEEE International Conference on Sensing, Communication, and Networking (SECON).

[35] Zhang X., Tang C., Yin K., Ni Q. (2021). Wifi-based cross-domain gesture recognition via modified prototypical networks. IEEE Internet Things J..

[36] Zheng Y., Zhang Y., Qian K., Zhang G., Liu Y., Wu C., Yang Z. Zero-effort cross-domain gesture recognition with wi-fi. Proceedings of the 17th Annual International Conference on Mobile Systems, Applications, and Services.

[37] Gao R., Zhang M., Zhang J., Li Y., Yi E., Wu D., Wang L., Zhang D. (2021). Towards position-independent sensing for gesture recognition with wi-fi. Proc. ACM Interact. Mob. Wearable Ubiquitous Technol..

[38] Goodfellow I.J., Pouget-Abadie J., Mirza M., Xu B., Warde-Farley D., Ozair S., Courville A., Bengio Y. Generative Adversarial Networks. Proceedings of the 27th International Conference on Neural Information Processing Systems.

[39] Qiu S., Liu L., Li J., Wang Z., Qin K., Jiang Y. Gaitsense: A potential assistance for physical rehabilitation by means of wearable sensors. Proceedings of the 2017 IEEE International Conference on Computational Science and Engineering (CSE) and IEEE International Conference on Embedded and Ubiquitous Computing (EUC).

[40] Li X., Chang L., Song F., Wang J., Chen X., Tang Z., Wang Z. (2021). Crossgr: Accurate and low-cost cross-target gesture recognition using wi-fi. Proc. ACM Interact. Mob. Wearable Ubiquitous Technol..

[41] Arjovsky M., Chintala S., Bottou L. Wasserstein generative adversarial networks. Proceedings of the 34th International Conference on Machine Learning.

[42] Zhang L., Wang Z., Yang L. Commercial wi-fi based fall detection with environment influence mitigation. Proceedings of the 2019 16th Annual IEEE International Conference on Sensing, Communication, and Networking (SECON).

[43] Zhang J., Tang Z., Li M., Fang D., Nurmi P., Wang Z. Crosssense: Towards cross-site and large-scale wifi sensing. Proceedings of the 24th Annual International Conference on Mobile Computing and Networking.

[44] Pan S.J., Yang Q. (2010). A survey on transfer learning. IEEE Trans. Knowl. Data Eng..

[45] Shi Z., Zhang J.A., Xu R.Y., Cheng Q. (2022). Environment-robust device-free human activity recognition with channel-state-information enhancement and one-shot learning. IEEE Trans. Mob. Comput..

[46] Ding S., Chen Z., Zheng T., Luo J. Rf-net: A unified meta-learning framework for rf-enabled one-shot human activity recognition. Proceedings of the 18th Conference on Embedded Networked Sensor Systems.

[47] Vaswani A., Shazeer N., Parmar N., Uszkoreit J., Jones L., Gomez A.N., Kaiser Ł., Polosukhin I. Attention is all you need. Proceedings of the 31st International Conference on Neural Information Processing Systems.

[48] Wang W., Liu A.X., Shahzad M., Ling K., Lu S. Understanding and modeling of wifi signal based human activity recognition. Proceedings of the 21st Annual International Conference on Mobile Computing and Networking.

[49] Gu Y., Zhang X., Wang Y., Wang M., Yan H., Ji Y., Liu Z., Li J., Dong M. (2022). Wigrunt: Wifi-enabled gesture recognition using dual-attention network. IEEE Trans. Hum.-Mach. Syst..

[50] Lin C., Ji C., Ma F., Wang L., Zhong W., Wu G. Wilca: Accelerating contactless authentication with limited data. Proceedings of the 2022 19th Annual IEEE International Conference on Sensing, Communication, and Networking (SECON).

[51] He K., Zhang X., Ren S., Sun J. Deep residual learning for image recognition. Proceedings of the 2016 IEEE Conference on Computer Vision and Pattern Recognition.

[52] Ioffe S., Szegedy C. Batch normalization: Accelerating deep network training by reducing internal covariate shift. Proceedings of the 32nd International Conference on International Conference on Machine Learning.

[53] Der Maaten L.V., Hinton G. (2008). Visualizing data using t-sne. J. Mach. Learn. Res..

[54] Finn C., Abbeel P., Levine S. Model-agnostic meta-learning for fast adaptation of deep networks. Proceedings of the 34th International Conference on Machine Learning.

[55] Kao C.-H., Chiu W.-C., Chen P.-Y. (2021). Maml is a noisy contrastive learner in classification. arXiv.

[56] Triantafillou E., Zhu T., Dumoulin V., Evci L.U., Xu K., Goroshin R., Gelada C., Swersky K., Manzagol P.-A., Manzagol P.A. (2019). Meta-dataset: A dataset of datasets for learning to learn from few examples. arXiv.

[57] Nichol A., Achiam J., Schulman J. (2018). On first-order meta-learning algorithms. arXiv.

[58] Radford A., Metz L., Chintala S. (2015). Unsupervised representation learning with deep convolutional generative adversarial networks. arXiv.

[59] Guo L., Wang L., Lin C., Liu J., Lu B., Fang J., Liu Z., Shan Z., Yang J., Guo S. (2019). Wiar: A public dataset for wifi-based activity recognition. IEEE Access.

